# The production effect in memory: multiple species of distinctiveness

**DOI:** 10.3389/fpsyg.2014.00886

**Published:** 2014-08-11

**Authors:** Michal Icht, Yaniv Mama, Daniel Algom

**Affiliations:** ^1^Department of Communication Disorders, Ariel UniversityAriel, Israel; ^2^Department of Behavioral Sciences, Ariel UniversityAriel, Israel; ^3^School of Psychological Sciences, Tel Aviv UniversityTel Aviv, Israel

**Keywords:** production effect, encoding distinctiveness, statistical distinctiveness, free recall, recognition

## Abstract

The production effect is the difference in memory favoring words read aloud relative to words read silently during study. According to a currently popular explanation, the distinctiveness of aloud words relative to silent words at the time of encoding underlies the better memory for the former. This distinctiveness is attributable to the additional dimension(s) of encoding for the aloud items that can be subsequently used during retrieval. In this study we argue that encoding distinctiveness is not the sole source of distinctiveness and that, in fact, there is an independent source of distinctiveness, statistical distinctiveness, which may or may not work in harmony with encoding distinctiveness in influencing memory. Statistical distinctiveness refers to the relative size of a subset of items marked by a(ny) unique property. Silently read words can carry statistical distinctiveness if they form a salient minority on the background of a majority of vocalized words. We show that, when the two sources are placed in opposition, statistical distinctiveness modifies the PE in a profound way.

## INTRODUCTION

The *production effect* (PE) documents the improvement in explicit memory for words that were produced aloud during study relative to words that were merely read silently (MacLeod, 2011). Recent research by MacLeod and his colleagues (e.g., MacLeod et al., 2010; Forrin et al., 2012; Ozubko et al., 2012) has not only revived interest in a little-known finding from the 70s and 80s of the last century (Hopkins and Edwards, 1972; Conway and Gathercole, 1987), but it also provided a more rigorous definition of the phenomenon and delineation of the conditions under which it does and does not occur. Everyday life entails possible instances of the PE in action. Students typically remember well those portions of the material that they had presented orally in class (but less well those portions merely summarized in their notebook). Rehearsals for stage productions typically start by oral reading of the respective texts by performers when silent reading would seem sufficient. Conversely, one often forgets an appointment despite writing down the date in the calendar (but the appointment is remembered if one mentions it, if unwittingly, to one’s spouse or a friend). How does one account for this robust phenomenon of memory, which is observed in the laboratory and everyday life alike?

A major idea driving much of the pertinent research has been that of *distinctiveness*: saying a word aloud provides for another process of encoding, making the word distinct against the backdrop of the other, silently read words. Our goal in the present study was to examine the conceptual underpinnings of the distinctiveness account. We show that this account actually entails a pair of separate definitions of the concept of “distinctiveness,” which may or may not agree with one another.

### TWO MEANINGS OF DISTINCTIVENESS

The idea that distinctiveness plays a facilitating role in learning and memory is hardly new. Its roots harken back to antiquity, to the pertinent theories of Plato and Aristotle, and, much later, to those of the British empiricists. In an early explication of the concept during the modern psychological era, Murdock (1960, p. 21) stressed the *relative* nature of distinctiveness such that “if there are no comparison stimuli, the concept of distinctiveness is simply not applicable.” Relative frequency was suggested as the main vehicle carrying distinctiveness in the frequency theory of learning and memory developed by Ekstrand et al. (1966, p. 567). These authors maintained that “the subjective difference in frequency of occurrence” serves as a major cue for learning and memory. The first study of the PE (Hopkins and Edwards, 1972) was similarly based on the notion of relative frequency as the mechanism at the root of the phenomenon (with vocal production construed as a frequency manipulation). Therefore, the meaning of distinctiveness espoused in the early period highlights its statistical nature: the fact that a subset of all items stands out as unique in some respect boosts memory for members of the subset.

In subsequent development, an almost imperceptible change in the meaning of distinctiveness has been taking place. Although the notion of relative frequency is retained (nominally, at the least; see the relational-distinctiveness account by Gathercole and Conway, 1988), it is increasingly superseded by the notion of the number of distinct encoding processes performed with the selected items. The greater the number of distinct processes afforded (vocalization, of course, but also encoding through activities such as writing, typing, or whispering), the greater the benefit to memory for those items. The approach is close in spirit to the proceduralist account of memory (Kolers and Roediger, 1984) and to research conducted within the framework of levels of processing (Craik and Lockhart, 1972; Jacoby and Craik, 1979). Closer still to the PE research, the benefits to memory wrought by augmented encoding is sometimes dubbed, the *distinctiveness heuristic* [Dodson and Schacter, 2001; see also, Hunt and McDaniel (1993), and Hunt and Worthen, 2006].

It is worth pausing to ponder the approach by Dodson and Schacter (2001) because it is instructive to appreciate the difference between encoding and statistical distinctiveness. In their model, decision processes evaluate the information retrieved in memory in order to attribute it to a source – old or new item. The participant assesses whether enough word information is remembered in order to respond that the item was seen. Notably, this process is performed with respect to the “distinctive” aloud items as the basis for judgment: the participant demands access to the aloud word information such that the absence of memory for this information indicates that the test item is new. At a first glimpse, Dodson and Schacter’s (2001) “distinctiveness heuristic” is compatible with statistical distinctiveness because the response to each item is decided on the basis of its distinctiveness. Closer scrutiny, however, reveals that the Dodson and Schacter’s (2001) model is merely a special case of encoding distinctiveness. The reason is that it is *always* the produced information that is distinctive in their model. The Dodson and Schacter’s (2001) approach is based on the assumption that people believe (in a “meta memorial belief”) that produced or otherwise activated information is distinctive and ought to be remembered better than non-distinctive information. In sharp contrast, the statistical distinctiveness approach asserts that *any* subset of items can be distinctive, produced or non-produced. The statistical approach is indifferent to the way that a subset of items is or becomes distinctive.

To recap, the currently popular perspective of diversity of production asserts that the record of a relatively rich encoding is preserved in memory so that it subsequently helps in deciding whether or not the item was studied. This meaning of distinctiveness refers thus to unique productions or depth of processing afforded to some of the items at study. The rich encoding is diagnostic with respect to the item’s status at study, conferring memory advantage on aloud items over silent items. Consequently, words studied aloud should always enjoy a memory advantage over words that were read silently due to the richer encoding of the former (e.g., Forrin et al., 2012). The statistical perspective, by contrast, asserts that *any* feature or *lack* of a feature that characterizes a subset of the items makes members of that subset distinctive, hence better recognizable. Consequently, it is possible that items read silently are better recognized than words said aloud when the former are distinctive (if by virtue of being a small minority).

Statistical distinctiveness was not a factor in the great bulk of past PE studies due to the trivial fact that the lists used were balanced across types of learning (50–50% of aloud and silent items in most studies, or 33–33–33%, and even 25–25–25–25% partitions of items in some studies). Clearly, the PE could not result from statistical distinctiveness in these studies. The downside, of course, was overlooking statistical distinctiveness as a potent modifier of the PE when one deviates from the balanced design. A main goal of the preset study was uncover this factor and contrast it with the traditional encoding distinctiveness account of the PE.

Because the two perspectives are not fully harmonious, statistical distinctiveness can be put in opposition to encoding distinctiveness. This was the tactic that we employed in the present study. Before turning to discuss the current tactic, however, we must consider briefly a recent alternative to the distinctiveness account, encoding, or statistical.

### MEMORY STRENGTH VERSUS DISTINCTIVENESS ACCOUNT OF THE PE

Although our study is concerned with distinctiveness, we would be remiss if we did not refer to recent developments that can pose a challenge to this account. The PE is typically obtained in a mixed-list, within-subject design. However, in a series of experimental studies augmented by sets of meta-analyses, Bodner and Taikh (2012), Fawcett (2013) and Bodner et al. (2014) obtained the PE in a between-subjects design in a recognition task. The point of departure for these studies is a cogent logical argument: whenever there is a performance difference between two conditions – memory for aloud vs. silent words – there is no *a priori* grounds to decide whether the difference reflects a benefit for the aloud words or a cost to the silent words. The PE is typically construed as a benefit to aloud items (MacLeod et al., 2010), but it can equally reflect a cost to silent items. Needed are control conditions entailing pure lists of silent-only and aloud-only items as a yardstick for comparison with the within-subjects effect. Implementing these controls, Bodner et al. (2014) found that the PE obtains with different groups of participants, each experiencing a pure list, and that the within-subjects PE mainly reflects costs suffered by the silent items.

The finding of a between-subjects PE (in addition to the typical within-subject PE) accords well with a memory strength account by which the more strongly encoded aloud words are remembered better regardless of mode presentation. This finding removes an important source of support for the distinctiveness account (the alleged restriction to a within-subjects design), but it does not invalidate it (Bodner and Taikh, 2012; Bodner et al., 2014). Distinctiveness still remains the currently best-supported account of the PE.

When considering memory strength, the discussions in the literature as well as the current summary refer to the rival distinctiveness account as diversity of encoding. Again, statistical distinctiveness was neither recognized nor tested. The separate-group lists of 100% aloud and 100% silent words as well as the within group lists of 50–50% silent–aloud words used are inappropriate for testing statistical distinctiveness. Because our goal in this study was to test species of distinctiveness, we used a within-subjects design. Nevertheless, our results do speak to the memory strength account. Moreover, there already exist data in the literature with pure lists that support the statistical distinctiveness account. These observations are best appreciated after considering the results of this study.

### PLACING STATISTICAL DISTINCTIVENESS IN OPPOSITION TO ENCODING DISTINCTIVENESS

Suppose that of a list of 60 words, 50 words are studied by vocalization, whereas the remaining 10 words are read silently. The two approaches to distinctiveness lead to conflicting predictions in this situation. Consider the encoding approach. Given that (a) the stimuli are presented in a mixed-list and that (b) vocalization affords for a further process of encoding, the produced words should be remembered better than those merely read silently. The statistical approach, by contrast, yields the opposite prediction. The minority of 10 words stand out as distinct against the backdrop of the other, produced words, the extra-processing of the latter notwithstanding. Given their distinctiveness, the minority of silently read words should be remembered better than the produced words.

We note that the tension between the two aspects of distinctiveness was recently noticed, if in an implicit manner, in a study by MacLeod et al. (2010). Espousing the encoding perspective, the authors wonder why the benefit of vocalization accrues to produced words “only when mixed with words read silently” especially as “remembering that a word was produced at study is diagnostic even if *all* the words were studied aloud” (p. 681; emphasis added). The authors attempt to resolve the anomaly by suggesting that people do not activate encoding context as a means for improving memory when all words are produced. Regardless of the plausibility of this argument, the situation depicted in our example would certainly suffice to trigger vocalization-produced advantage.

In this study, we make the contrast between the two sources of distinctiveness explicit, pitting one source against the other. Our goal was to pry apart the contribution (if any) of each source. Therefore, we tested memory in situations similar to that depicted in our example. Our point of departure was a mixed-list in which a random half of the words are produced – the standard preparation in the PE literature. Note again that this standard condition removes relative frequency as a cue for distinctiveness and, consequently, as a source for the PE. Notably, in two additional conditions we departed from the common 50–50% frequency situation. In one condition, the majority of the words on the list were produced, whereas in the other condition the majority of the words were read silently. According to the perspective that attributes the PE to richness of encoding, memory for the produced words should be superior in* all* three conditions (recall that the list is mixed in all conditions). According to the statistical or relative frequency perspective, words in the smaller subset (whether or not produced) will be better remembered due to their uniqueness.

## MATERIALS AND METHODS

### PARTICIPANTS

Sixty men and women, undergraduate students from Ariel University, received course credit for performing in the experiment. There were 13 men and 47 women, their age ranging between 18 and 32 years (mean age: 23 years). All gave their informed consent to take part in the study, which was approved by the ethics committee. Each participant was randomly assigned to one of three learning conditions, differing in the relative frequency of vocally produced words. In the first condition, half of the words were read aloud, whereas the remaining half was read silently. In the second condition, 20% only of the words were vocally produced, with the rest, a majority of 80%, read silently. In the third condition, 80% of the words were produced and the remaining 20% read silently. Twenty participants performed in each learning condition.

### APPARATUS AND STIMULI

The pool of items consisted of 80 common Hebrew words, bi-syllabic nouns, three to five letters long, with frequencies of greater than 12 per million (Frost and Plaut, 2005). From this pool, 40 words were selected for study, a different sample for each participant. The remaining 40 items served as distractor items in the subsequent recognition test.

During study, each word was presented singly for view. The word appeared at the center of a 15 inch color monitor (Compaq laptop computer under control of Direct-RT program). The words were presented in black (28-point Arial), against a white background. On each trial, a small icon (2 cm^2^) appeared approximately 5 cm above the study word. The icon entailed a small picture of an eye or of a microphone. The icon indicated the appropriate mode of production for that word: the eye indicated silent reading, the microphone vocal production.

### DESIGN

#### Study

In each of the three conditions, the 40 study words were randomly divided into two subsets defined by the requested mode of learning. One subset was learned by vocal production and the other subset was learned by silent reading. The size of each subset differed across conditions. Aloud words (signaled by the microphone icon) comprised 50% of the items in the first condition, 20% in the second condition, and 80% in the third condition. The size of the subset of silent words (signaled by the eye icon) varied in a complementary fashion.

#### Filler task

A brief task of number generation followed the study phase. The participants were instructed to vocally produce numbers between 1 and 9 during a time window of 4 min.

#### Memory: free recall

Each participant was asked to write down from memory as many study words as she or he could recall. An empty sheet of paper and a pencil was provided by the experimenter.

#### Memory: recognition

This test followed free recall. A list of 80 words, half from study and half unstudied distractors, were presented singly for view. The participant made a simple binary decision, old (from study), or new (unstudied), for each word by pressing the appropriate key. Viewing conditions were the same as in the study phase except for the absence of icons. Responding was not speeded. The next word appeared 400 ms after the response.

### PROCEDURE

The participants were tested individually in a dimly lit room. Upon arrival, each participant was assigned to one of three learning conditions in a random fashion. The participant was seated at a distance of 60 cm from the center of the screen. The participant was told that the goal was to learn each word via the mode signaled by the icon (eye, microphone) and that tests of memory would follow the presentation of the words. Each word was presented for 3 s followed by 1 s blank screen. After the presentation of the 40 words, the participants performed in a short (filler) task, generating numbers between 1 and 9 in a random fashion for 4 min. Free recall followed (without explicit time constraint), performed by writing down as many study words from memory as possible. Finally, in recognition, the participants made a simple old–new decision with respect to each of 80 words (40 from study, 40 new) without emphasis on speed (which was not recorded).

## RESULTS

**FIGURE 1:**
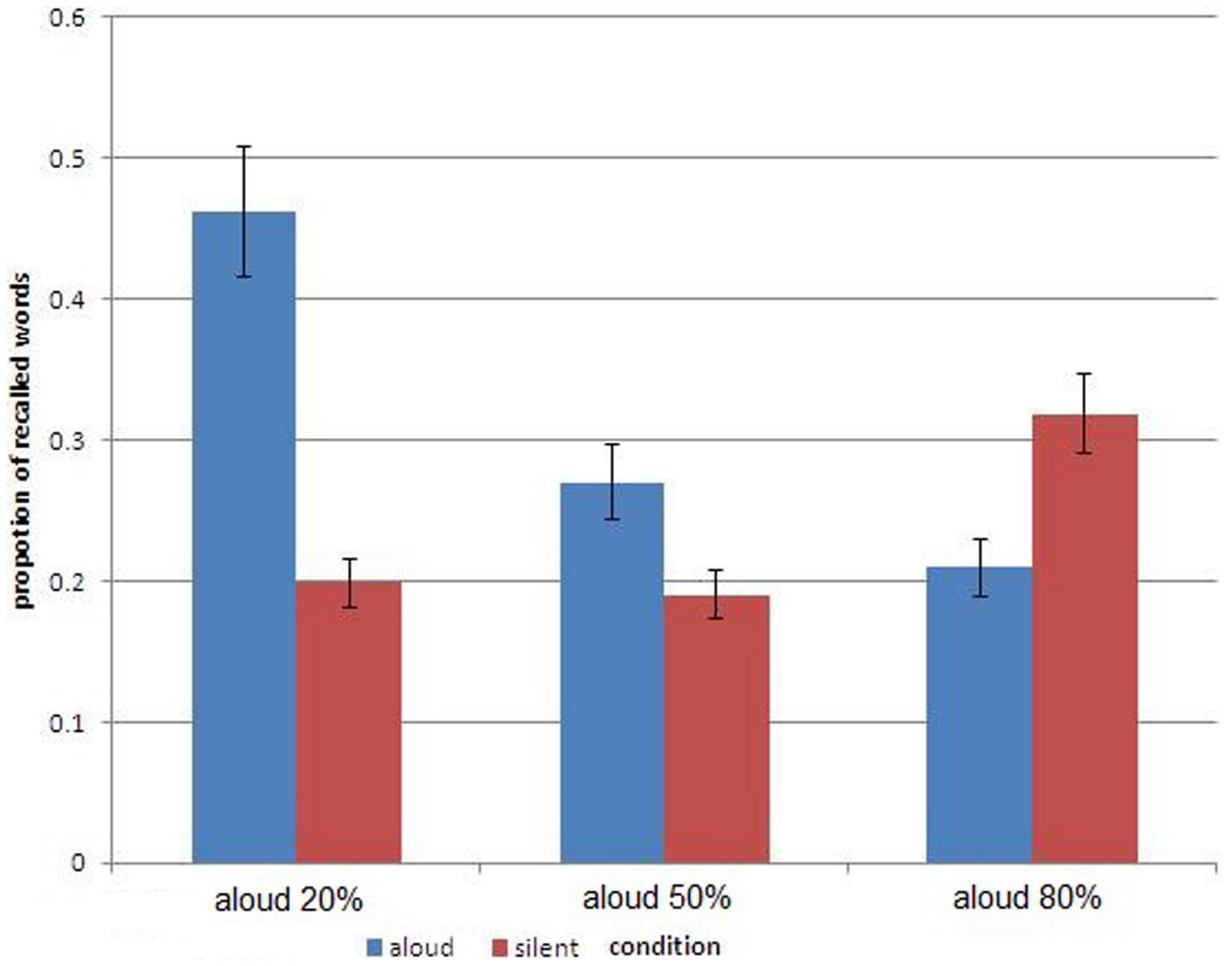
**Percentage of recalled words, calculated separately for the subsets of aloud and silent words, in three groups defined by the size of the subset of the aloud words.** The bars represent one standard error around the mean.

**Figure 1** gives the results of free recall. Plotted is percent recall for aloud and for silent words in three groups defined by the size of the subset of the aloud (or silent) words. Consider first the results obtained in the group presented with the list in which 50% of the words were said aloud (with the other 50% read silently). Clearly, the aloud words were recalled better. This PE is entirely attributable to encoding distinctiveness: memory for the vocalized words benefited from the extra-processing in learning. Note that, in this common design, statistical distinctiveness does not play a role due to the fact that silent reading and vocalization come in even numbers.

Consider next the learning condition depicted at the left of **Figure 1** in which only 20% of the words were vocalized (with the remaining 80% read silently). Again, there is a PE, documenting the memory advantage accrued to vocally produced words as opposed to words that were read silently. However, what is most revealing about the results of this group is the size of this advantage: the current PE was four times its size in the 50–50% group. We attribute the large PE to the joint effect of encoding distinctiveness and of statistical distinctiveness. The vocalized words were distinctive due to the unique process of production in encoding but they were also distinctive due to their dissimilarity to the majority of the items, standing out as a salient minority. Note also that the increase in PE came almost solely due to the improved memory for the vocalized items.

Finally, consider the condition depicted at the right of **Figure 1** in which 80% of the words were produced and only 20% were read silently. This condition pits encoding distinctiveness against statistical distinctiveness. The former favors vocalized words, the latter silent words. A glimpse at **Figure 1** shows that statistical distinctiveness proved dominant. We recorded a reverse PE by which memory for silently read words was better than memory for vocalized words. The statistical distinctiveness conferred on silent words by virtue of comprising a discernible minority proved a stronger boost to memory than that provided by encoding distinctiveness to vocalized words.

Statistical analysis supports the conclusions based on visual inspection of **Figure 1**. A mixed-measures ANOVA with subset-size (20, 50, 80% aloud) as a between-subjects variable and type of learning (vocal production, silent reading) as a within subjects variable revealed a significant interaction between these main factors [*F*(2,57) = 17.795, *p* < 0.0001]. The interaction supports the reversal of the PE depicted in **Figure 1**. The difference in memory between aloud and silent words was significant in each of the three learning conditions. In the 50–50% group, participants remembered words that were read aloud better (27%) than words which were read silently (19%), *t*(19) = 2.018, *p* < 0.05. In the 20% aloud group, the advantage of read-aloud words over silently read words was larger (46.2 and 19.8%, respectively), *t*(19) = 6.66, *p* < 0.001. Finally, in the 80% aloud group, the pattern reversed with silently read words remembered better than words that were produced aloud (31.8 and 20.9%, respectively), *t*(19) = 2.091, *p* < 0.05.

Augmenting the previous analyses, we also performed one-way ANOVAs across the three conditions separately for aloud and silent words. Both analyses indicated significant differences in recall in the three groups [*F*(2,57) = 18.85, *p* < 0.01, and *F*(2,57) = 3.91, *p* < 0.05, for aloud and silent words, respectively]. The boost to memory of the aloud items when they formed a small minority is underscored by comparing directly the 20% versus 50% aloud conditions [*t*(38) = 4.17, *p* < 0.01]. The boost to memory of silent items when *they* formed a minority is sustained by the direct comparison of performance between the 80 aloud and the 50% aloud conditions [*t*(38) = 2.37, *p* < 0.05]. Moreover, the mean memory performance in each group was mimicked in the individual data. Thus, 15 of the 20 participants in the 50–50% group showed the pattern of advantage for aloud words. Similarly, 19 of the 20 participants in the 20% aloud condition followed the pattern of the mean data. Most striking, 14 of the 20 participants in the 80% aloud condition displayed the PE reversal evident in the group data.

**FIGURE 2:**
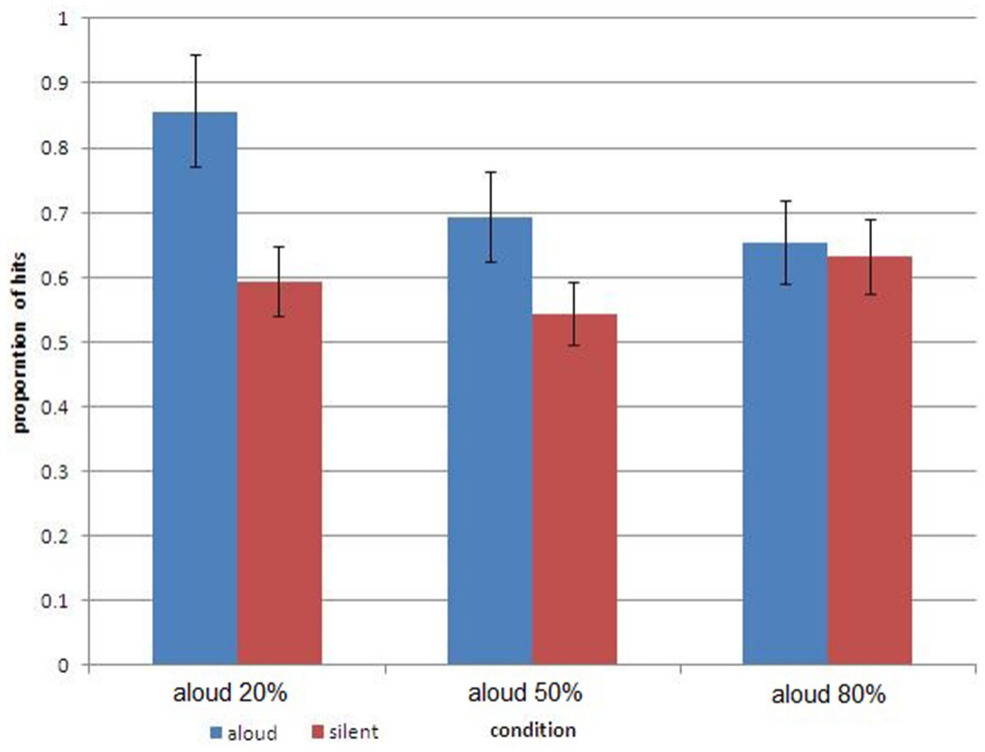
**Percentage of correctly recognized words, calculated separately for the subsets of aloud and silent words, in three groups defined by the size of the subset of aloud words.** The bars represent one standard error around the mean.

We present the results of recognition in **Figure 2**. We must be a bit circumspect when evaluating these results because recognition was administered after testing free recall so that all sorts of carryover effects cannot be ruled out. Nevertheless, the outcome of recognition is suggestive.

Inspection of **Figure 2** with respect to recognition reveals much of the same trend observed in **Figure 1** with respect to free recall. Memory performance for vocalized words increased as the relative frequency of these words in the list decreased. As a result, the PE observed in the standard 50–50% condition increased in the 20% aloud condition, but evaporated in the 80% aloud condition. We note though that this variation in PE was produced by different rates of recognition of the vocalized words, not by those of the silent words. In fact, recognition of the latter remained largely invariant across conditions.

Statistical analysis supports these conclusions. A mixed-measures ANOVA with subset-size (20, 50, 80% aloud) as a between subjects variable and type of learning (vocal production, silent reading) as a within subjects variable revealed again a significant interaction between the two main factors [*F*(2,57) = 7.55, *p* < 0.001]. The interaction supports the “dilution” of the PE depicted in **Figure 2**. In the 50–50% group, participants recognized correctly words that were read aloud better (69.2%) than they did words that were read silently (54.2%), *t*(19) = 3.961, *p* < 0.001. In the 20% aloud condition, the advantage for read-aloud over silently read words was even greater (85.6 and 59.3%, respectively), *t*(19) = 6.379, *p* < 0.001. The PE vanished altogether in the 80% aloud condition with comparable recognition of aloud and silent words (65.3 and 63.1%, respectively), *t*(19) = 0.444, *p* > 0.3. Again, the mean memory performance in each group faithfully reflected in individual data. Thus, 16 of the 20 participants in the 50–50% group showed an advantage for aloud words, and 19 of the 20 participants in the 20% aloud condition followed the pattern of the mean data. A one-way ANOVA for the aloud words revealed significant differences in recognition across the three groups [*F*(2,57) = 7.68, *p* < 0.01], but the same analysis did not yield significant results for the silent words [*F*(2,57) = 1.24, *P* > 0.05]. The boost to recognition memory for aloud items when they comprise a minority is reinforced by the direct comparison of the 20% versus 50% aloud items conditions [*t*(38) = 2.61, *p* < 0.01].

## DISCUSSION

The results of this study demonstrated once again the robustness of the PE under standard conditions of learning. Our results also support a distinctiveness explanation for the PE. This much granted, the study shows that there are (at least) two separate sources of distinctiveness that may or may not act in harmony with one another. By underscoring the dual roots of distinctiveness, we also qualify the conditions under which the PE or a reversed PE appears. Our results demonstrate that statistical distinctiveness is as powerful as is encoding distinctiveness in influencing memory.

If statistical distinctiveness or relative frequency is so powerful, why has it been ignored in existing PE research? Our account implicates the standard design used: equal numbers of items are always allocated to the different types of processing deployed in learning. When two types of processing are tested (silent reading versus vocalization), the list of items is divided up evenly between the two; when a third type is considered (silent reading, vocalization, and whispering), the list is sliced into three equal portions. This practice rules out a role for *relative* frequency. Earlier research (Ekstrand et al., 1966) has shown relative frequency to be a powerful mnemonic device. Our study reinforces its role in generating the PE (see more on the role of item segregation in memory in the recent study by Icht et al., 2013).

Our goal in this study was to demonstrate the potency of statistical distinctiveness. Nevertheless, as we recounted, the results speak to the memory strength account as well. The reversal of the PE observed in our study rules against a memory strength account to the same extent that it does against the encoding distinctiveness account. We must be circumspect though as, numerically, the reversal entailed eight items only. Replication and extension are clearly invited in future research.

Because we did not attempt to test a memory strength account here, our study did not include a between-subjects pure lists manipulation. Fortunately though, the critical data do exist in the literature. The original experiments and data are construed along different lines, of course, but, upon new scrutiny augmented by the present results, they support statistical distinctiveness. In Experiments 1 and 2 of the Ozubko and MacLeod (2010) study, different groups of participants were presented with an all aloud or an all silent list of words, respectively. Subsequently, the participants in each group were presented with a mixed-list entailing 50–50% of aloud and silent words. The task for the participants was a source memory test in which they were asked to identify which list each word came from. The critical point to note is that, considering the two lists together, there is a clear minority (or majority) of either aloud or silent words. Although each list is balanced onto itself, the combined outcome is a minority of 50 silent words against 150 aloud words in one condition and a minority of 50 silent words against 150 aloud words in the other condition. The statistical distinctiveness account predicts that memory performance is superior with the minority words regardless of their mode of learning, aloud or silent. In particular, the minority of silent words should be better remembered than the majority of aloud words despite the stronger encoding of the latter. This is precisely what Ozubko and MacLeod (2010) have found. A glimpse at their summary Table 1 (p. 1545) reveals the same reversal of the PE observed in the present study. These results pose a challenge to the encoding distinctiveness account as well as to a memory strength account.

The validity of the memory interpretation of the Ozubko and MacLeod (2010) results has been challenged in the recent study by Bodner and Taikh (2012). These authors argue that the source memory task is subject to biases based on the specific composition of each list. Recognition memory cannot be based on such attributions. Moreover, Bodner and Taikh (2012) found a reverse PE when relative frequency was controlled (Experiment 2) and they found the typical PE when the aloud items were more frequent than silent ones (Experiment 3). One should realize though that statistical distinctiveness is not the sole source of the PE (so it did not produce the reverse PE in Experiment 2). The task in Experiment 3 of the Bodner and Taikh (2012) study differed considerably from the main task of free recall used in the present study and it differed even from the usual recognition task. The reservations and results of Bodner and Taikh (2012) granted, we still find the pattern obtained by Ozubko and MacLeod (2010) strongly suggestive of statistical distinctiveness.

We must also mention a new account of the PE proposed recently by Jonker et al. (2014). According to their idea, based on the item-order account by McDaniel and Bugg (2008), inter-item order guides recall of silent items more than it does that of aloud items due to the encapsulated nature of the unusual encoding accorded to the latter. In mixed-lists in particular, the order-based memory of silent items is disrupted more than memory for aloud items because memory for the latter relies much less on order. This account predicts different amounts of disruption of silent items in our 20, 50, and 80% aloud conditions. In particular, silent items are expected to suffer less disruption in the 20% aloud group in which they comprise a large majority. Consequently, the PE is expected to be smallest in this group. Our results violate this prediction so that they are inconsistent with item-order theory.

What is the mechanism underlying the effect of statistical distinctiveness? We believe that the root cause is the enhanced information value of rare stimuli. Common stimuli are not surprising and hence less informative. According to a basic tenet of information theory (Garner, 1962, 1974; Baird and Noma, 1978; Melara and Algom, 2003), the smaller the a priori probability of a stimulus, the greater the information provided by its appearance. Consequently, each word in the smaller subset carries more information than each word in the larger subset. Larger amounts of information command extra attention, which, in turn, fosters learning and improves subsequent memory.

We mentioned the relatively small number or words involved in the reversal or evaporation of the PE, which reminds one of the list-length effect (Roberts, 1972): when the number of words in the list increases, the number of words recalled increases although their proportion decreases. This effect was present in the two biased conditions of our study and should be given greater attention in future research. In particular, in the condition with the majority of silently read words the raw number of silent words recalled was greater than that of the aloud words recalled (although the proportion of aloud words recalled was much greater as is evident in **Figure 1**). This result documents (in a convoluted way to be sure) that the advantage of produced words subsumed under the PE is context dependent and can be easily reversed.

Finally, let us relate to the two test administered, free recall and recognition. In the present study, statistical distinctiveness engendered a reverse PE in free recall. We did not observe a full reversal in recognition, although the absence of the PE in the 80% aloud condition is suggestive. The recognition data are less decisive due to the fact that recognition was administered after free recall.

In sum, the present results disclosed a hitherto hidden source of distinctiveness that also contributes to the PE when dealing with unbalanced lists. This source can act in tandem with the typically discussed source of encoding uniqueness or in opposition to that source. An unresolved question awaiting future investigation is the relative salience (Melara and Algom, 2003) of the two sources of distinctiveness. What distribution of study words suffices to engender the statistical effect? How different should the procedures in learning be in order to engender the encoding effect? In the meantime, investigators should be on the watch for statistical distinctiveness, especially when using non-random allocation of items in encoding. Notably, too, one cannot rule out additional species of distinctiveness beyond the two considered in this study [see Mama et al. (2013), for the role of various species of salience in cognition]. A final worthy task for investigators is to determine the relationship between statistical distinctiveness and memory strength accounts especially in view of the newly found malleability of the PE.

## Conflict of Interest Statement

The authors declare that the research was conducted in the absence of any commercial or financial relationships that could be construed as a potential conflict of interest.
